# Supplementary material for: Multi‐dataset identification of innovative feature genes and molecular mechanisms in keratoconus
Source: J Cell Mol Med. 2024 Sep 19;28(18):e70079. doi: 10.1111/jcmm.70079 (PMC11412914; doi:10.1111/jcmm.70079)
Supplement: Supplementary file 2 — Material S2. [file JCMM-28-e70079-s002.zip › 7.GSEA analysis/4.MYRF/KEGG_LEISHMANIA_INFECTION.html]

Details for gene set KEGG\_LEISHMANIA\_INFECTION[GSEA]

|  || Dataset | MYRF.group.cls#h\_versus\_l |
| Phenotype | group.cls#h\_versus\_l |
| Upregulated in class | l |
| GeneSet | KEGG\_LEISHMANIA\_INFECTION |
| Enrichment Score (ES) | -0.53508663 |
| Normalized Enrichment Score (NES) | -1.5201571 |
| Nominal p-value | 0.040733196 |
| FDR q-value | 0.20333254 |
| FWER p-Value | 0.735 |
Table: GSEA Results Summary

  

Fig 1: Enrichment plot: KEGG\_LEISHMANIA\_INFECTION      
 Profile of the Running ES Score & Positions of GeneSet Members on the Rank Ordered List

  

| SYMBOL | TITLE | RANK IN GENE LIST | RANK METRIC SCORE | RUNNING ES | CORE ENRICHMENT || 1 | CR1 | na | 1481 | 0.402 | -0.0156 | No |
| 2 | ITGA4 | na | 1618 | 0.389 | 0.0068 | No |
| 3 | IL12A | na | 1851 | 0.369 | 0.0250 | No |
| 4 | ELK1 | na | 3767 | 0.262 | -0.0126 | No |
| 5 | NCF2 | na | 5163 | 0.217 | -0.0381 | No |
| 6 | NCF4 | na | 6934 | 0.166 | -0.0780 | No |
| 7 | HLA-DOA | na | 8167 | 0.134 | -0.1045 | No |
| 8 | MAPK12 | na | 8572 | 0.125 | -0.1077 | No |
| 9 | FCGR1A | na | 8801 | 0.120 | -0.1061 | No |
| 10 | IRAK1 | na | 10013 | 0.096 | -0.1346 | No |
| 11 | MAPK11 | na | 10606 | 0.085 | -0.1460 | No |
| 12 | MAPK13 | na | 10951 | 0.079 | -0.1506 | No |
| 13 | FCGR2A | na | 11470 | 0.071 | -0.1608 | No |
| 14 | MAPK14 | na | 11965 | 0.064 | -0.1707 | No |
| 15 | STAT1 | na | 12606 | 0.055 | -0.1854 | No |
| 16 | CYBA | na | 12687 | 0.054 | -0.1841 | No |
| 17 | MAPK3 | na | 14382 | 0.034 | -0.2307 | No |
| 18 | JUN | na | 16671 | 0.011 | -0.2959 | No |
| 19 | TAB1 | na | 19282 | -0.014 | -0.3703 | No |
| 20 | JAK1 | na | 20051 | -0.022 | -0.3910 | No |
| 21 | HLA-DRB5 | na | 20183 | -0.024 | -0.3931 | No |
| 22 | MYD88 | na | 20189 | -0.024 | -0.3916 | No |
| 23 | MAPK1 | na | 20406 | -0.027 | -0.3961 | No |
| 24 | IRAK4 | na | 20511 | -0.028 | -0.3972 | No |
| 25 | FOS | na | 20589 | -0.029 | -0.3975 | No |
| 26 | ITGB2 | na | 21824 | -0.044 | -0.4301 | No |
| 27 | TGFB2 | na | 22177 | -0.048 | -0.4371 | No |
| 28 | TAB2 | na | 22545 | -0.053 | -0.4441 | No |
| 29 | IL12B | na | 22624 | -0.054 | -0.4427 | No |
| 30 | JAK2 | na | 23139 | -0.062 | -0.4534 | No |
| 31 | HLA-DRB3 | na | 23156 | -0.062 | -0.4496 | No |
| 32 | TRAF6 | na | 23526 | -0.068 | -0.4557 | No |
| 33 | HLA-DQB1 | na | 24038 | -0.076 | -0.4653 | No |
| 34 | HLA-DRB1 | na | 24046 | -0.077 | -0.4603 | No |
| 35 | HLA-DOB | na | 24105 | -0.078 | -0.4567 | No |
| 36 | MAP3K7 | na | 24724 | -0.088 | -0.4686 | No |
| 37 | IFNGR1 | na | 25005 | -0.093 | -0.4704 | No |
| 38 | NCF1 | na | 25156 | -0.096 | -0.4682 | No |
| 39 | NFKBIB | na | 25462 | -0.102 | -0.4701 | No |
| 40 | PTPN6 | na | 25517 | -0.103 | -0.4647 | No |
| 41 | PRKCB | na | 25770 | -0.108 | -0.4647 | No |
| 42 | HLA-DMA | na | 27498 | -0.148 | -0.5045 | No |
| 43 | NFKB1 | na | 28241 | -0.168 | -0.5146 | No |
| 44 | FCGR3A | na | 28556 | -0.177 | -0.5118 | No |
| 45 | IL10 | na | 28824 | -0.186 | -0.5069 | No |
| 46 | IFNGR2 | na | 29802 | -0.217 | -0.5204 | Yes |
| 47 | ITGB1 | na | 29873 | -0.219 | -0.5076 | Yes |
| 48 | TLR2 | na | 29913 | -0.220 | -0.4939 | Yes |
| 49 | HLA-DRB4 | na | 30247 | -0.232 | -0.4878 | Yes |
| 50 | RELA | na | 31135 | -0.271 | -0.4951 | Yes |
| 51 | FCGR2C | na | 31311 | -0.280 | -0.4812 | Yes |
| 52 | MARCKSL1 | na | 31314 | -0.280 | -0.4624 | Yes |
| 53 | NFKBIA | na | 31525 | -0.292 | -0.4487 | Yes |
| 54 | ITGAM | na | 31711 | -0.302 | -0.4336 | Yes |
| 55 | TLR4 | na | 31882 | -0.311 | -0.4176 | Yes |
| 56 | C3 | na | 31953 | -0.315 | -0.3983 | Yes |
| 57 | HLA-DPA1 | na | 32053 | -0.320 | -0.3796 | Yes |
| 58 | PTGS2 | na | 32511 | -0.351 | -0.3690 | Yes |
| 59 | TGFB1 | na | 32868 | -0.377 | -0.3538 | Yes |
| 60 | IL1A | na | 32896 | -0.380 | -0.3289 | Yes |
| 61 | TGFB3 | na | 33024 | -0.389 | -0.3063 | Yes |
| 62 | HLA-DRA | na | 33031 | -0.390 | -0.2802 | Yes |
| 63 | IL4 | na | 33045 | -0.391 | -0.2541 | Yes |
| 64 | IFNG | na | 33150 | -0.402 | -0.2300 | Yes |
| 65 | HLA-DMB | na | 33256 | -0.414 | -0.2051 | Yes |
| 66 | NOS2 | na | 34080 | -0.537 | -0.1925 | Yes |
| 67 | HLA-DQA2 | na | 34217 | -0.576 | -0.1575 | Yes |
| 68 | IL1B | na | 34248 | -0.586 | -0.1188 | Yes |
| 69 | TNF | na | 34328 | -0.609 | -0.0800 | Yes |
| 70 | HLA-DPB1 | na | 34331 | -0.609 | -0.0389 | Yes |
| 71 | HLA-DQA1 | na | 34602 | -0.745 | 0.0036 | Yes |
Table: GSEA details [plain text format]

  

Fig 2: KEGG\_LEISHMANIA\_INFECTION      
 Blue-Pink O' Gram in the Space of the Analyzed GeneSet

  

Fig 3: KEGG\_LEISHMANIA\_INFECTION: Random ES distribution      
 Gene set null distribution of ES for **KEGG\_LEISHMANIA\_INFECTION**
